# Seaman Prize Awarded

**Published:** 1905-11

**Authors:** 


### Seaman Prize Awarded.

At the fourteenth annual meeting of the Association of Military Surgeons of the United States, which opened in Detroit on September 27, Major Jefferson Randolph Kean, U. S. A., of Washington, D. C., was announced to be the winner of the Seaman prize for 1905. This is a prize of $500 offered for the best essay on "The Prevention of Disease in the Army and the Best Method of Accomplishing that Result." Major Kean's paper contained a plea for the re-establishment of the canteen at army posts, and he showed by statistics that since the abolishing of the canteen all kinds of diseases traceable to dissipation have increased among the soldiers of the regular army.

A TWENTY-DOLLAR-A-YEAR advertiser wants a ten-dollar reading notice free, each month. I wrote him:

"The waiter shouted down the hall—
We don't give bread with one codfish ball."

That's all I said.

DR. J. W. KENNEY, of San Antonio, has one of the best equipped and best managed establishments in the State for surgical cases and a special annex for cases of drug addiction. His treatment for the morphine mania is a modification of the hyoscine method, first publicly given the profession by Dr. M. K. Lort (TEXAS MEDICAL JOURNAL, November, 1901), who was formerly associated with him. I take pleasure in recommending Dr. Kenney to the profession of the State.

